# The impact of community care services on older people’s psychological health: an empirical study in Liaoning Province, China

**DOI:** 10.3389/fpubh.2023.1199830

**Published:** 2023-08-04

**Authors:** Qin Sen, Zhang Lei

**Affiliations:** School of Humanity and Law, Northeastern University, Shenyang, China

**Keywords:** community care service, psychological health, population aging, structural equation model, heterogeneity analysis

## Abstract

**Background:**

Rapid population aging in China means it is imperative to establish a comprehensive care service system for older people. Currently, China is vigorously promoting the development of community care services for older people which should, ideally, focus on psychological health in addition to physical health. This study examined the impact of community care services on older people’s psychological health.

**Methods:**

Survey data (*n* = 741) were collected from people aged 60 years and older in Liaoning Province, China, in which various community care services for older people were provided. Information was collected regarding the types of services provided (e.g., meal services, medical and social care), participants’ demographic details (age, gender, economic circumstances, etc.), and their psychological health (e.g., loneliness, life satisfaction). The impact of the various care services on older people’s psychological health was subsequently examined through the construction of a structural equation model.

**Results:**

Community care services for older people had a significant positive impact on their psychological health, with the most significant positive impact on cultural and sports activities, visiting and chat services, and emotional counseling. The impact of community care services on sub-groups of older people (e.g., those who were disabled, socially isolated and/or poor) was different.

**Conclusion:**

It is necessary to provide comprehensive and high-quality community care services, organize diverse cultural, sports, and recreational activities, provide differentiated and specific services for older people, and formulate corresponding service guidelines.

## 1. Introduction

Since 2000 China has experienced rapid aging of its population and is a trend that is predicted to continue well into the future (1). According to the seventh national population census data, by the end of 2020, 18.70% of the population in China will be aged 60 years and older. The “Fourteenth Five-Year Plan” of the National Plan for the Development of the Aged Cause and the Care Service System clearly emphasizes the need to continue to expand the “balanced, reasonable, high-quality, efficient, urban and rural coverage of care services, benefiting the entire population.” At present, family pensions, community pensions and institutional pensions are the three main types of urban pension models in China, and a “9,064” pension service model has been formed (90% of the older adults live at home, 6% live in community, and 4% live in institutions). This shows that community care services play a crucial role in China’s pension system (2). As an old industrial base province in Northeast China, Liaoning Province ranks first in terms of population aging due to the influences of industrial structures, economic levels, social culture and other factors. Since 2020, Liaoning Province has taken the lead in carrying out reform pilot work of community care services for older people in China. A series of policies and regulations have been promulgated, including the Regulations of Shenyang Municipality on Home-based Care Services. It has carried out reform pilot work of community care services for older people in 200 communities in nine cities, including Shenyang and Dalian, and formed a series of working models with demonstration effects.

With the trend of aging, the health of the older population is of concern. Older people tend to suffer from more physical and mental diseases and are prone to psychological problems, inducing the risk of suicide (3). According to 2018 Report on Older Adults’ Psychological Health in China, 95% of older people have different degrees of psychological disorders, and they are prone to emotional problems. In June 2019, the National Health Commission also pointed out that changes in living conditions, social relations and physical conditions associated with older age, such as the weakening of the body and the death of a partner, bring negative feelings of inferiority and worthlessness. If these feelings are not appropriately managed, psychological problems such as depression and/or anxiety may result. According to the World Health Organization, “health is not only the absence of disease or weakness in the body but also the physical, psychological and social integrity of a person.” The psychological health of older people is not only a medical and health problem but also a public problem affecting social stability and development (4). At present, child support, institutional care and community care services focus on meeting the survival and physiological needs of older people, while overlooking or neglecting their spiritual needs and psychological problems (5).

Recently, the Chinese government has placed greater importance on the issue of the care of older people in policies, focusing on their living conditions, and demanding the improvement of the quality of care services to meet their diverse needs. However, while most policies tend to guarantee basic living needs, such as food, housing, and basic medical care, and emphasis on resource support for service institutions, there is a lack of support for psychological services.

Similarly, current research on community care services mainly focuses on which service items should be established (6), how processes and quality can be improved (7), and how older people’s satisfaction with services can be enhanced (8). However, there is limited research on the impact of community care services on older people’s health, especially their psychological health, and there is a lack of investigation into the demand for services and service effectiveness, which directly affects the improvement and development of community care services. From this point of view, the psychological health of older people in China receives little attention but requires consideration.

Given this, this study is committed to studying, within the context of actively promoting community care services for older people in China, whether the community can become the real spiritual home for older people, provide them with high-quality and convenient services, and thus positively impact their psychological health. An additional question was whether this impact differs for sub-groups of the population. This is an important issue affecting the construction and improvement of the community care service system for older people in China. This study explored the impact of community care services on the psychological health of older people using the research results of 200 community care pilots for older people in Liaoning Province.

## 2. Literature review

### 2.1. Community care services for older people

Population aging and the care of older people are social issues faced by all countries globally. Welfare countries have implemented models of community care for older people since the 1960s. In the UK, the Department of Health and Social Security (DHSS) proposed that “use community facilities and resources to provide long-term care and assistance for people in need of care in the community, especially the very vulnerable older adults “, and an important objective of community care services is to reduce loneliness and abandonment experienced by older people. Ayers and Lyman (9) emphasized the critical role of psychological counseling and emotional assistance in caring for older people. Connelly (10) divides the content of community care into four types, the first is life care (including home services, apartments, etc.), the second is material support (including providing food, tax relief, etc.), the third is medical support (including disease treatment, health care, etc.), and the fourth is overall care (including improving the living environment, promoting social participation, etc.).

### 2.2. Definition of psychological health

The World Federation for Mental Health (WFMH) defines psychological health as “developing one’s mood into the best state within the range of physical intelligence and emotion that is not inconsistent with other people’s mental health.” The World Health Organization (WHO) (2014) summarizes three characteristics of psychological health: “good personality, good ability to handle affairs, and good interpersonal relationship (11).” Wu (12) reported that as older people experience physical aging and social role changes, their psychological health also presents different characteristics, and attention should be paid to their cognitive abilities and adaptability.

### 2.3. Measurement of psychological health

In terms of psychological health measurement, early researchers mainly analyzed older people’s psychological health from a single perspective of subjective psychological feelings. Siebert et al. (13) reported that loneliness, depression, subjective well-being and life satisfaction are sensitive indicators that can be used to quantify older people’s mental health. Kozma and Stones (14) developed the Memorial University Newfoundland Scale of Happiness (MUNSH), which is based on the theory of emotional balance and studies the mental health of older people from the dimensions of loneliness, depression and subjective well-being. The Geriatric Depression Scale GDS and the Center for Epidemiological Studies Depression Scale CES-D are also common tools for measuring the degree of depression and mental health of older people (15, 16). In China, Li et al. (17) from the Institute of Psychology of the Chinese Academy of Sciences compiled the “Mental Health Scale for the Older People (Urban Version),” and identified five dimensions of psychological health: cognitive efficacy, emotional experience, self-awareness, interpersonal communication and adaptability. Based on literature research, consulting case analysis, expert interviews and discussions, and open-ended questionnaires, Deng and Zheng (18) developed the “Mental Health Scale for the Urban Middle-aged and older Adults,” which involves loneliness, depression, cognition, and interpersonal relations, etc.

Based on the research results of scholars from China and other countries, the measurement of the psychological health of older people using subjective emotional indicators only is limited. A comprehensive evaluation index system including loneliness, cognitive ability, life satisfaction and interpersonal activity participation as the main dimensions should be established.

### 2.4. Interfering factors of psychological health of older people

In order to fully understand the root causes of psychological problems and diseases of older people and improve their psychological health, scholars from all countries are committed to analyzing the factors that influence their psychological health, which can be roughly summarized into three aspects: physiological condition (sleep quality, illness, etc.), social relationships (family relationships, interpersonal relationships, etc.), and economic conditions (income levels, social security). Regarding physiological factors, Jorunn (19) studied the relationship between the ability to perform Activities of Daily Living (ADL) and loneliness in older people through structured interviews in a rehabilitation center, and concluded that ADL functions could reduce loneliness. Jacobs et al. (20) analyzed the relationship between physical health and loneliness in older people and found that those with physical dysfunction were more likely to feel lonely. In terms of social relationships, Wu (21) found that the social support of family, neighbors and friends affects the subjective well-being and mental health of older people by affecting their self-esteem, and reducing loneliness. Regarding economic conditions, Saber (22) reported that the level of economic income significantly impacts older people’s happiness index.

## 3. Methods

### 3.1. Sample data sources

The data for this study were derived from the results of a survey conducted by the Institute of Urban and Rural Community Construction of Northeast University in 200 community care service pilot communities in Liaoning Province from 2021 to 2023. The survey adopted a multi-stage stratified cluster sampling method and was aimed at people aged 60 years and older. Step 1: The sample size was determined to be 800 people by calculation; Step 2: The sample size for each city was determined on the basis of the size of the population in each of the 14 cities in Liaoning Province; Step 3: Sampling communities were identified using a systematic random sampling method; Step 4: Using cluster sampling method, six samples of older people in each community were randomly selected and asked to complete a survey. A total of 852 questionnaires were distributed, and 741 valid questionnaires were returned, yielding a response rate of 87%. The data have strong universality and representativeness.

### 3.2. Variable selection and measurement

Independent variables: community care services for older people. According to the relevant regulations of the Ministry of Civil Affairs and other departments, community care services for older people include eight elements: living care services, meal and cleaning services, medical and health services, visiting and chat services, emotional counseling services, emergency rescue services, cultural and sports activities services, and legal aid services. Values were assigned to each of service item for the purpose of analysis, with a value of ‘1’ assigned if the community provided this social service, while a value of ‘0’ was assigned if the service was not provided.

Dependent variables: older people’s psychological health. According to the measurements of psychological health in the literature review, this paper aimed to build a comprehensive and objective measurement index of older people’s psychological health. Therefore, the four indicators of loneliness, cognitive ability, life satisfaction and participation in activities were selected to measure older people’s psychological health. “Loneliness” is a sensitive indicator of psychological health with which it is significantly negatively correlated, and the higher an individual’s psychological health, the lower the perceived loneliness (23). The question “Do you often feel lonely?” was included in the questionnaire which was answered using a 1–5 scale where “1 = always, 2 = often, 3 = sometimes, 4 = rarely, and 5 = never.” “Cognitive ability” refers to the ability of the human brain to process, store and extract information. Based on the Mental Health Scale for the Older Adults (Urban Version), this study designed seven questions that reflected seven aspects: memory, spatial recognition, learning ability, understanding ability, expression ability, calculation ability and reaction speed. Each answer was assigned the value of “1 = correct, 0 = wrong or unknown,” and the scores of the seven questions were added to compute a total score. “Life satisfaction” is a key indicator of older people’s subjective well-being and Song et al. (24, 25) showed that the life satisfaction index of older people could be used to reflect their psychological health status. Life satisfaction was measured by the question “Are you satisfied with your current life?” which was answered using a 1–5 scale where: “1 = very dissatisfied, 2 = dissatisfied, 3 = average, 4 = satisfied, and 5 = very satisfied.” “Participation in activities” belongs to the dimension of interpersonal communication, which is measured by the frequency of engagement in social activities. Scholars from all countries regard interpersonal activities as a measure of older people’s psychological health. “Participating in activities” was assessed by the question “Do you participate in the following activities now?” including “music or dance, sports, playing cards or mahjong, communicating with friends, voluntary activities, and other outdoor activities.” The question was answered using a 1–5 scale where: “1 = not participating, 2 = not every month but at least once a year, 3 = not every week but at least once a month, 4 = not every day but at least once a week, and 5 = participating almost every day.” The answers to the six items were summated to compute a total score.

Control variables: referring to the existing research about the factors that influence older peoples’ mental health, data were collected regarding the participant’s personal characteristics, behavior habits, economic status and family status, including age (1 = 60–65 years old, 2 = 65–70 years old, 3 = 70–75 years old, 4 = 75–80 years old, 5 = 80 and over), gender (0 = female, 1 = male), living conditions (1 = family, 2 = living alone, 3 = nursing home), marital status (1 = married and living with a spouse, 2 = married but not living with spouse, 3 = divorced, 4 = widowed, 5 = unmarried), physical exercise (0 = no physical exercise in the past, 1 = physical exercise in the past), disability (1 = no disability, 2 = mild disability, 3 = moderate disability, 4 = severe disability) and economic situation (1 = very poor, 2 = relatively poor, 3 = average, 4 = relatively wealthy, 5 = very wealthy).

### 3.3. Empirical models

#### 3.3.1. Confirmatory factor analysis

In this study, four variables: loneliness, cognitive ability, life satisfaction and participation activities were used as indicators of “older people’s psychological health.” In contrast “older people’s psychological health” was a latent variable, which is the common part of the four measurement indicators – a common factor. In order to test whether the whole factor model was significant, confirmatory factor analysis (CFA) was conducted on the four indicators of loneliness, cognitive ability, life satisfaction and participation in activities to verify whether the factor load coefficient of “older people’s mental health” was significant, and to verify whether the measurement of “older people’s mental health” was valid.

#### 3.3.2. Structural equation model (SEM)

As a confirmatory method, the structural equation model can explore the structural relationship between variables, and is widely used in psychology, sociology and other fields (26, 27). In this study, the explanatory variable “community care service” was an explicit variable, and the explanatory variable “older people’s psychological health” was a latent variable measured by four indicator variables: loneliness, cognitive ability, life satisfaction, and social activities. The structural model equation of community care services and older people’s psychological health is shown by the formula (1):


(1) η=α+ΓX+ζ

The measurement model is shown in formula (2):


(2) y=γη+ε

In formula (1), η Is a latent variable, indicating older people’s mental health; Γ Indicates the change of the latent variable after each unit change of the indicator variable; X refers to community care services that affect older people’s psychological health, which are composed of living care services, meal and cleaning services, medical and health services, visiting and chat services, emotional counseling services, emergency rescue services, cultural and sports activities services and legal aid services; ζ Represents a random perturbation term. In the formula (2), y = (y1, y2, y3, y4) is a group of indicator variables for older people’s psychological health. This study used four indicators: loneliness, cognitive ability, life satisfaction, and social activities; ε indicates the measurement error item (Table 1).

**Table 1:** Sample size allocation.

City	Amount of sample communities per city	Amount of samples per community	Sample size
Shenyang	30	6	78
Dalian	20	6	72
Anshan	11	6	60
Chaoyang	10	6	60
Jinzhou	10	6	60
Huludao	9	6	60
Tieling	9	6	60
Yingkou	8	6	54
Dandong	7	6	60
Fushun	6	6	60
Fuxin	6	6	54
Liaoyang	6	6	60
Panjin	5	6	60
Benxi	5	6	54
**Sum**	142		842

## 4. Analysis of empirical results

### 4.1. Descriptive statistical results

Descriptive data and participants’ characteristics are summarized in Table 2 which shows that the average score for loneliness was 4.03, indicating that respondents had a low sense of loneliness as a whole and do not feel particularly lonely. Cognitive ability is composed of memory, spatial recognition, learning ability, understanding ability, expression ability, calculation ability and reaction speed, and the average score was 6.52, indicating that respondents had high overall cognitive ability and normal intelligence. The average life satisfaction score was 3.88, indicating that participants were relatively satisfied with their current life as a whole and had a positive and optimistic attitude toward life. However, the average number of people participating in activities was 9.63, which indicates that participants had less contact with people and were not fully involved in various social activities. According to the social withdrawal theory, people’s abilities will inevitably decline with increasing age, and they gradually lose their social roles, thus reducing social interaction and participation in activities. This view is consistent with the results of this research.

**Table 2:** Characteristics of the participants and availability of support services.

	Variables	*N*	Means	Standard deviation	Minimum values	Maximum values
Control variable	Age	738	2.12	1.037	1	5
	Gender	736	0.42	0.303	0	1
	Living conditions	723	1.22	0.455	1	3
	Marital status	707	2.77	1.476	1	5
	Physical exercise	728	0.69	0.454	0	1
	Disability	719	1.38	0.762	1	4
	Economic situation	711	1.96	0.629	1	5
Dependent variables	Loneliness	717	4.03	0.996	1	5
	Cognitive ability	689	6.52	1.084	0	7
	Life satisfaction	718	3.88	0.796	1	5
	Participation activities	723	9.63	3.830	6	30
Independent variables	Living care services	702	0.12	0.303	0	1
	Meal and cleaning services	726	0.83	0.317	0	1
	Medical and health services	714	0.57	0.475	0	1
	Visit and chat services	722	0.15	0.353	0	1
	Emotional counseling services	706	0.17	0.287	0	1
	Emergency rescue services	715	0.28	0.464	0	1
	Cultural and sports activities services	710	0.37	0.412	0	1
	Legal aid services	721	0.21	0.493	0	1

In terms of community care services, the average value of each service is ranked from high to low as follows: meal and cleaning services, medical and health services, cultural and sports activities services, emergency rescue services, legal aid services, emotional counseling services, visit and chat services, and living care services, with the average value of 0.83, 0.57, 0.37, 0.28, 0.21, 0.17, 0.15, and 0.12, respectively. Overall, the service items and supply coverage of the existing community care services are seriously insufficient, with the focus on physical health and basic living needs while social and spiritual needs are overlooked. According to Maslow’s hierarchy of needs theory, community care services should not only focus on the first level of physical needs and the second level of safety needs, but also focus on the third and fourth levels of social and respect needs.

### 4.2. Model estimation results

**Figure 1:**
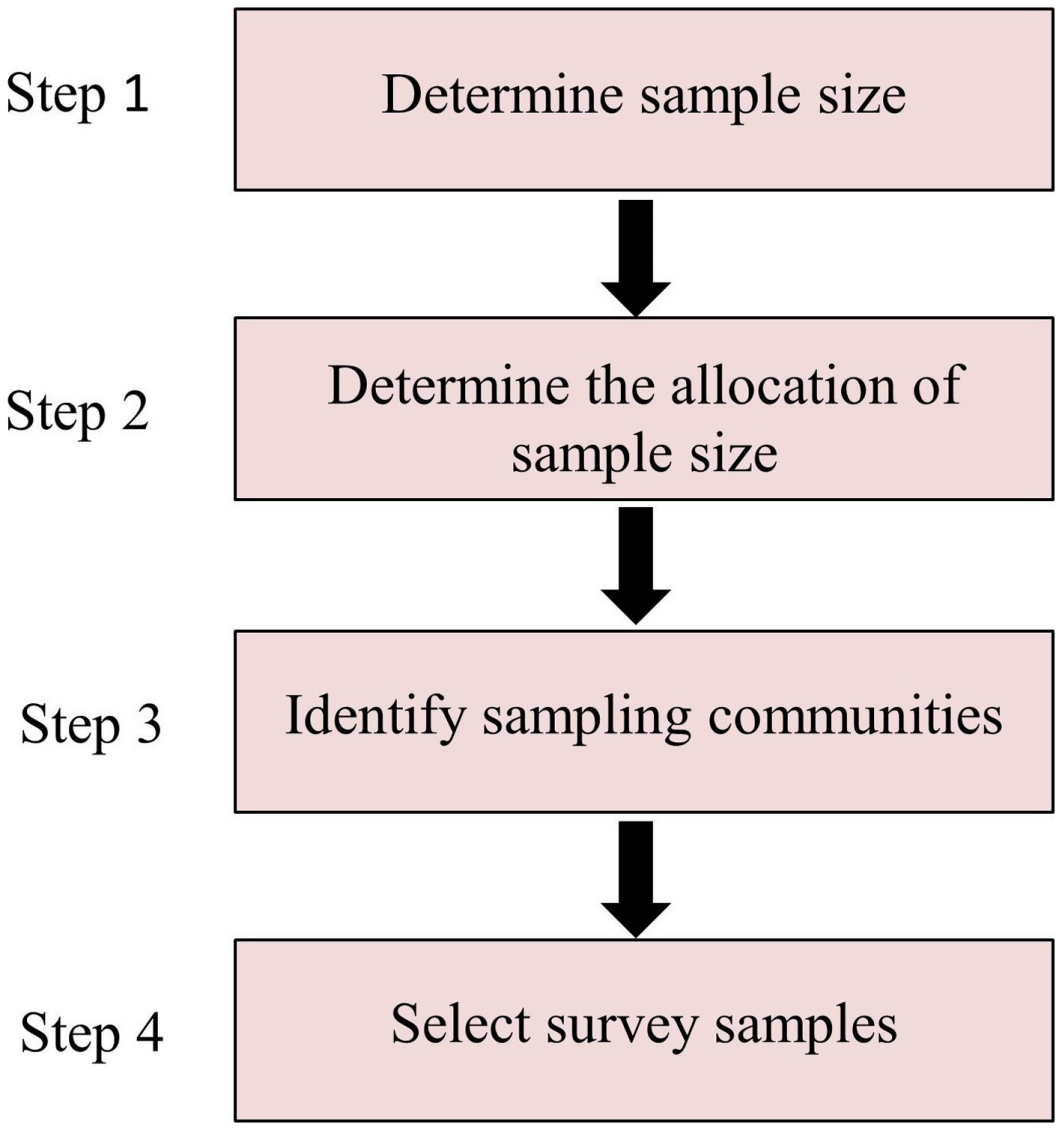
Chart of sample selection.

Before running the model, the data in this study were first tested for reliability using SPSS software. The results showed that the Cronbach’s α of each study variable was above 0.6, indicating that the data had high reliability. Subsequently, AMOS software was used to draw a model path map and perform data operations, and after debugging, the model was optimized. The final output is shown in Figure 1. The fitting index RMSEA = 0.067 and SRMR = 0.039 indicates that the model fitness of the constructed factor model was good.

According to the model estimation results in Figure 1, community care services have a significant impact on older people’s psychological health with an impact coefficient of 0.72. The three services with the most significant impact were cultural and sports activity services, visiting and chat services, and emotional counseling services, with coefficients of 0.38, 0.26, and 0.22, respectively. This result indicates that within the community, the involvement of professional social workers and psychological counselors, carrying out diverse cultural and sports activities to enhance the social participation and communication circle of older people can enrich their leisure life, provide a sense of collective belonging and existence, and maintain a positive and healthy mentality. Visiting and chat services are a direct way to comfort people. Service personnel can play the role of relatives or friends of empty nesters to a certain extent and alleviate older people’s loneliness through communicating with them. At the same time, due to personal, family, or neighborhood factors, older people are prone to suffer from depression and anxiety. Counseling services provided by professional psychological consultants can help older people reduce internal misunderstandings and distress and lower their risk of mental illness.

**Figure 2:**
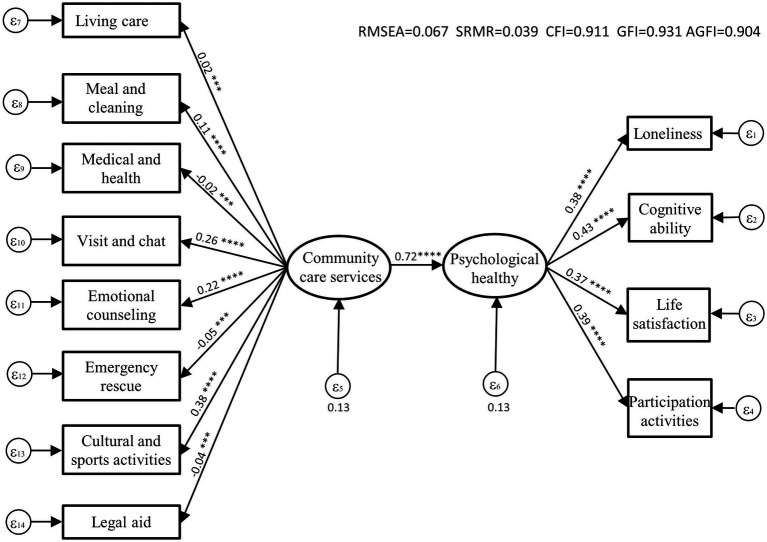
Path model of the impact of community care services on older people’s psychological health.

In addition to these three items, the service items that ranked fourth to fifth in terms of impact were: meal and cleaning services and living care services. These service categories can improve the quality of life of older people, ensure that they live with dignity in their familiar community environment, thereby making them feel the meaning and value of life, and enhancing their life satisfaction. While the other three service elements - medical and health services, emergency rescue services and legal aid services were found to have no significant effects, and may even have a negative impact on older people’s mental health. This is mainly due to the relatively short development time of the three services, which are still in the initial stage of development, insufficient experience of practitioners, high service costs, and insufficient coverage, resulting in a lower overall service level (Figure 2).

### 4.3. Robustness check

Considering that older people’s psychological health is also affected by other factors, this study also added control variables to the following models (1) and (2), including age, gender, living conditions, and whether participants undertook physical exercise. The regression coefficients and significant changes in the models were evaluated to assess the robustness of the empirical models.

In Table 3, Part A shows the estimated results of the structural model for various factors affecting older people’s mental health, and Part B shows the estimated results of the measurement model, which is essentially consistent with the estimated results of the structural equation model. Part C shows the fitting indicators for the entire model, and of them, RMSEA<0.08, SRMR<0.05, and *R*^2^ ≈ 0.7312, indicating that the overall fit of the model is good, and the empirical results are robust and acceptable.

**Table 3:** Estimation results of extended SEM Model.

Model	Equation	(1)	(2)
	Estimation method	ML	ML
A structural model	Living care	0.018^**^	0.015^**^
		(1.30)	(0.72)
	Meal and cleaning	0.153^***^	0.148^***^
		(3.08)	(3.21)
	Medical and health	−0.025^**^	−0.019^**^
		(−2.26)	(−2.34)
	Visit and chat	0.027^**^	0.034^**^
		(2.21)	(2.58)
	Emotional counseling	0.045^***^	0.076^**^
		(3.77)	(3.30)
	Emergency rescue	−0.023^**^	−0.031^**^
		(−1.32)	(−0.72)
	Cultural and sports activities	0.421^****^	0.395^****^
		(3.30)	(3.43)
	Legal aid	0.018^**^	0.019^**^
		(1.45)	(1.37)
	Age	−0.009^****^	−0.009^****^
		(−15.01)	(−16.31)
	Gender	0.036^****^	0.036^****^
		(4.64)	(4.60)
	Living conditions		−0.012^**^
			(−1.54)
	Physical exercise		0.189^****^
			(15.80)
B measurement model	Loneliness	0.355^****^	0.412^****^
		(2.738)	(2.145)
	Cognitive ability	1.569^****^	1.450^****^
		(6.099)	(5.730)
	Life satisfaction	0.996^****^	0.610^****^
		(3.194)	(3.935)
	Participation activities	8.326^****^	7.689^****^
		(11.124)	(10.517)
	N	734	721
C fit indices	RMSEA	0.072	0.072
	SRMR	0.036	0.034
	*R^2^*	0.814	0.772

****indicates *p* is significant at 0.1%, ***indicates *p* is significant at 1%, and **indicates *p* is significant at 5%.

### 4.4. Heterogeneity analysis

The data were further analyzed to assess heterogeneity and whether the impact of community care services on older people’s psychological health varied according to whether they live alone, whether they are disabled, and their economic level.

Table 4 shows the results of the heterogeneity analysis. Results showed that living care services have a differential impact on disabled versus non-disabled older people, and have a significant positive impact on the psychological health of disabled older people, but no significant impact on non-disabled older people. The reason is that disabled people are unable to complete daily life independently due to physical impairments, and need the help of professional service personnel (28). Meal and cleaning services were also found to have a differential impact on the psychological health of solitary older people, disabled older people, and poor older people. This is likely due to the greater vulnerability of these groups who have a greater need for these services. In addition, the coverage and quality of this service are currently high, and older people can obtain it in a convenient and low-cost manner, thereby improving their life satisfaction. Furthermore, older people can benefit from the opportunity of interacting and communicating with others during meals which is likely to reduce their sense of loneliness (29). Medical and health services were found to have a positive impact on the psychological health of non-solitary older people, disabled older people, and wealthy older people. By comparison, the impact on other groups of older people was negative. This may be due to disabled older people having a stronger demand for medical services, requiring family support to a certain extent, and relatively high cost (30). Visiting and chat services have a differential impact on those who live alone and have a significant positive impact on their psychological health. This is most likely because solitary older people lack the care of their families and children and are eager to communicate with others (31). Emotional counseling services have a positive impact on the psychological health of different groups of older people, particularly the solitary and the disabled, who are more vulnerable to psychological problems due to life stresses. Emergency rescue services have a positive impact on the psychological health of the solitary and disabled older people, while their impact on other groups of older people was negative. This may be because solitary and disabled older people have impaired physical functioning and may be more likely to face the risk of sudden illness and/or accidental injury. Emergency rescue services provide protection for older people through remote monitoring, improving their sense of security. Cultural and sports activities services have a significant positive impact on the psychological health of all groups of older people, indicating that regardless of residential, physical, and economic status, older people have a clear need to participate in various social interaction activities to reduce loneliness, improve life satisfaction, social skills, and maintain their cognitive abilities. Legal aid service has a negative impact on the psychological health of non-solitary and poor older people, while it has a positive impact on other groups of older people. This may be because, when faced with legal disputes, the non-solitary group typically has children to assist them, while those who are poor may have limited understanding of the legal system, and generally resolve disputes through other means.

**Table 4:** Heterogeneity analysis results.

Variables	Items	Solitary older adults	Non solitary older adults	Disabled older adults	Non disabled older adults	Wealthy older adults	Poor older adults
Living care	SE	0.037	0.016	0.051	0.016	0.027	0.019
	*Z*	3.14	1.79	2.35	1.96	2.79	5.06
	Significance	0.024	0.007	0.029^****^	0.011	0.013	0.026^***^
Meal and cleaning	SE	0.032	0.021	0.056	0.018	0.049	0.018
	*Z*	4.32	1.44	3.23	1.84	2.79	0.58
	Significance	0.075^****^	0.026^**^	0.142^****^	0.058^**^	0.024	0.028^***^
Medical and health	SE	0.044	0.013	0.05	0.018	0.039	0.011
	*Z*	−1.08	2.14	1.75	−0.38	1.82	−0.33
	Significance	−0.012	0.073^**^	0.052^**^	−0.027	0.013^***^	−0.006
Visit and chat	SE	0.061	0.017	0.052	0.025	0.041	0.028
	*Z*	1.5	1.3	1.48	3.34	3.41	1.5
	significance	0.079^****^	0.034^**^	0.077^**^	0.084^**^	0.142^**^	0.042
Emotional counseling	SE	0.042	0.073	0.125	0.048	0.325	0.198
	*Z*	5.23	2.75	4.96	1.77	3.28	2.66
	Significance	0.275^****^	0.112^***^	0.189^***^	0.063^**^	0.137^***^	0.158^***^
Emergency rescue	SE	0.048	0.022	0.046	0.019	0.051	0.013
	*Z*	3.24	−1.86	1.09	−1.27	−2.78	−1.03
	Significance	0.074^**^	−0.029	0.035^**^	−0.093	−0.034	−0.012
Cultural and sports activities	SE	0.058	0.015	0.045	0.022	0.04	0.024
	*Z*	2.86	6.73	0.8	6.58	0.15	3.36
	Significance	0.166^***^	0.103^****^	0.036	0.146^****^	0.012	0.082^****^
Legal aid	SE	0.047	0.012	0.041	0.018	0.037	0.019
	*Z*	1.48	−1.05	0.21	0.59	2.78	−1.04
	Significance	0.07	−0.034	0.008	0.048^**^	0.054	−0.039
*N*		126	597	96	623	226	485

****indicates *p* is significant at 0.1%, ***indicates *p* is significant at 1%, and **indicates *p* is significant at 5%.

Overall, community care services significantly impact the psychological health of the solitary, disabled and poor older people, proving that these vulnerable groups have a greater need for community care services. At the same time, different types of services have different effects on different groups of older people. Those who are solitary need more spiritual services, the disabled need more life support services, and the poor need more cultural and entertainment services. This analysis result is consistent with other Chinese scholars’ research reports. For example, Wu (21) reported that solitary older people lack the support and care of their families regarding life and spirituality and need social interventions to improve their psychological health. Huang (32) also reported that disabled older people need customized and specific services based on daily living and care services, but the current supply is still significantly insufficient. Wang (33) reported that poor older people have fewer opportunities to participate in social activities and created the phenomenon of “conceptual poverty” and “spiritual poverty.” It is necessary to encourage and support poor older people to participate in community activities and improve their quality of life.

## 5. Discussion

This study examined the impact of community care services on older people’s psychological health in Liaoning Province. Overall, community care services have a significant positive impact on older people’s psychological health, which is consistent with previous research (34).

This study further indicates that only five of the eight service items have a positive impact, while the remaining three have a negative impact, including medical and health services, emergency rescue services, and legal aid services. Previous studies have not found this phenomenon, but some Chinese scholars have pointed out that these three services have obvious shortcomings such as insufficient supply and poor professionalism, and thus cannot meet the needs of older people (35), which confirms the conclusion of this study.

In addition, this study indicates that living conditions, disability, and older people’s economic circumstances have a differential impact on the results. Community care services have a greater impact on the psychological health of older people living alone, the disabled, and the poor. This indicates that vulnerable groups need more customized and high-quality community services which has also been confirmed by other relevant studies.

In China, the “Healthy Aging” policy is committed to improving the comprehensive health of older people, especially their psychological health and social adaptability. This study is based on the policy background of “healthy aging,” and differs from other studies that only focus on physical health. Instead, it focuses on multidimensional psychological health, to test the effectiveness of different community care services. This study fills the research gap in this field and suggests directions for Chinese community care services to address issues associated with population aging.

At the same time, however, this study is limited to Liaoning Province, which has a very large aging population, and does not cover the whole country, which may result in the research results not being widely representative. In the future, with further research, more comprehensive conclusions may be drawn.

## 6. Conclusion

This study was based on survey data from pilot programs of community care services in Liaoning Province, to explore the impact of community care services on the psychological health of older people. The empirical results show that, of the eight service elements stipulated by policy, the implementation of five types of services had a significant positive impact on older people’s psychological health, namely, cultural and sports activities services, visiting and chat services, emotional counseling services, meal and cleaning services, and living care services, while the other three service elements - medical and health services, emergency rescue services and legal aid services were found to have no significant effects, and may even have a negative impact on older people’s mental health. This is mainly due to the relatively short development time of the three services, which are still in the initial stage of development, insufficient experience of practitioners, high service costs, and insufficient coverage, resulting in a lower overall service level. In addition, there are differences in the results of community care services under different living conditions, disability conditions, and economic conditions. It is necessary to focus on the psychological health of older people living alone, the disabled, and the poor, and provide specific community care services for these groups.

In response to these conclusions and to the policy spirit of “healthy aging” and “active aging” in China, the following suggestions are proposed to improve the community-based elder care model, and improve the psychological health status of the older population:

First, provide comprehensive and high-quality community care services for older people, focusing on improving the quality and coverage of spiritual services. Research shows that with the development of China’s economy and society, older people’s needs are characterized by diversity. After basic physiological and survival needs are met, emotional satisfaction, the acquisition of dignity, and the establishment of social interaction are equally important. From the perspective of the supply side of care services, existing care services for older people are characterized by “emphasizing physiology but neglecting psychology.” The coverage of spiritual services is relatively low, and the service level is relatively poor. It is recommended that the development of spiritual services for older people in the community be strengthened through fund allocation, talent cultivation, facility construction, team cooperation, and project introduction.

Second, build a social activity platform for older people, and organize diverse cultural, sports, and other entertainment activities for them. Of the eight community care service elements, cultural and sports activities had the most significant impact on older people’s psychological health, which requires lower capital costs in comparison to other services. In the case of limited community resources, priority could be given to the implementation of recreational activities and services. Communities should establish activity centers for older people, fully mobilize their human resources, and regularly organize activities for them, thereby connecting them to the community and enriching their lives.

Third, it is important to focus on the psychological health of the most vulnerable and disadvantaged groups of older people, i.e., those who are solitary, disabled and/or poor, and give them more respect and care. Generally speaking, while impairments in physical functioning are obvious and receive attention, loneliness and lack of social interaction are more difficult to detect but are likely to result in loneliness, depression and an increased risk of mental illness. Therefore, when providing care services for older people, it is important to provide targeted services based on the particular circumstances of those who are particularly vulnerable, and develop corresponding service guidelines.

## Data availability statement

The original contributions presented in the study are included in the article/supplementary material, further inquiries can be directed to the corresponding author.

## Ethics statement

The studies involving human participants were reviewed and approved by Northeastern University Ethics Committee. The patients/participants provided their written informed consent to participate in this study.

## Author contributions

All authors listed have made a substantial, direct, and intellectual contribution to the work and approved it for publication.

## Conflict of interest

The authors declare that the research was conducted in the absence of any commercial or financial relationships that could be construed as a potential conflict of interest.

## Publisher’s note

All claims expressed in this article are solely those of the authors and do not necessarily represent those of their affiliated organizations, or those of the publisher, the editors and the reviewers. Any product that may be evaluated in this article, or claim that may be made by its manufacturer, is not guaranteed or endorsed by the publisher.
